# The relationship of the carriership of allelic variations in rs2228145 (A > C) of the IL6R gene with the levels of VCAM1 and ICAM1 gene transcripts in patients with essential hypertension

**DOI:** 10.18699/VJ20.600

**Published:** 2020-02

**Authors:** L.V. Topchieva, V.A. Korneva, I.V. Kurbatova

**Affiliations:** Institute of Biology of the Karelian Research Centre of the Russian Academy of Sciences, Petrozavodsk, Russia; Petrozavodsk State University, Petrozavodsk, Russia; Institute of Biology of the Karelian Research Centre of the Russian Academy of Sciences, Petrozavodsk, Russia

**Keywords:** interleukin 6, IL6R gene, essential arterial hypertension, endothelial dysfunction, интерлейкин 6, ген IL6R, эссенциальная артериальная гипертензия, эндотелиальная дисфункция

## Abstract

The levels of plasma interleukin 6 and its soluble receptors were found to be elevated in subjects with cardiovascular diseases, which points to amplification of the IL-6-mediated trans-signaling pathway in cells and the development of chronic inflammation. The allelic variation in the rs2228145 IL6R gene is associated with a change in the contents of the soluble and membrane-bound receptor forms mediating the biological activity of IL-6. Cytokine IL-6 is involved in the development of endothelial dysfunction by regulating the expression of the VCAM1 and ICAM1 genes, encoding intercellular adhesion molecules. Prior to this work, no data on the association of essential arterial hypertension (EAH) with rs2228145 allelic variations of the IL6R gene have been reported. The aim of our work was to study the relationship of the carriership of rs2228145 (A > C) allelic variations with the development of EAH and the VCAM1 and ICAM1 transcript levels. We analyzed samples of DNA isolated from the whole blood of 148 healthy donors and 152 patients with EAH (stages I–II). The genotyping was performed by PCR-RFLP. The level of transcripts in peripheral blood leukocytes (PBL) was assessed by real-time PCR. Differences in the frequency distributions of rs2228145 (A > C) genotypes between the control group and the group of patients with EAH (χ2 = 9.303) were found. The frequency of the CC genotype in EAH patients was higher than in healthy people (0.191 and 0.095, respectively). The risk of EAH (I–II stages) development was shown to be 2.3 times higher in CC genotype carriers as compared to individuals with other genotypes (OR = 2.257, 95 % confidence interval 1.100–4.468). The levels of VCAM1 and ICAM1 gene transcripts in PBL of patients with EAH were significantly higher than in healthy people. The level of ICAM1 gene transcripts was almost 4 times higher in patients with CC genotype. The Kruskal–Wallis analysis of variance revealed an effect of rs2228145 (A > C) genotype on the transcriptional activity of ICAM1, which argues for its role in the pathogenesis of endothelial dysfunction and essential hypertension.

## Abstract (ru)

При сердечно-сосудистых заболеваниях в плазме крови наблюдается повышение содержания интерлейкина 6 и его растворимых рецепторов, что указывает на усиление IL-6/sIL-6R сигнала в клетках и развитие хронического воспаления. Носительство аллельных вариаций по rs2228145 гена IL6R ассоциировано с изменением содержания растворимой и мембраносвязанной форм рецептора, опосредующих биологическую активность самого цитокина. IL-6 участвует в развитии эндотелиальной дисфункции посредством регуляции экспрессии генов VCAM1 и ICAM1, кодирующих молекулы межклеточной адгезии. До настоящей работы данные об ассоциации эссенциальной артериальной гипертензии (ЭАГ) с аллельными вариациями по rs2228145 гена IL6R не были представлены. Цель исследования – изучить связь носительства аллельных вариаций по rs2228145 (A > C) с развитием ЭАГ и уровнем транскриптов генов VCAM1, ICAM1. Для этого нами использованы образцы ДНК, выделенной из цельной крови здоровых доноров (148) и пациентов с ЭАГ (I–II стадии) (152). Генотипирование проводили методом ПЦР-ПДРФ. Уровень транскриптов в лейкоцитах периферической крови оценивали с помощью ПЦР в режиме реального времени. Обнаружены различия в распределении частот генотипов по rs2228145 (A > C) в контрольной группе и группе пациентов с ЭАГ (χ2 = 9.303). Частота генотипа СС в группе больных людей оказалась выше, чем в группе здоровых (0.191 и 0.095 соответственно). Выявлено, что у носителей генотипа СС риск развития ЭАГ (I–II стадии) в 2.3 раза выше (ОШ = 2.257, 95 % доверительный интервал 1.100–4.468), чем у лиц, имеющих альтернативные генотипы. Уровень транскриптов генов VCAM1, ICAM1 значимо выше в лейкоцитах периферической крови больных ЭАГ, чем здоровых людей. Содер- жание транскриптов гена ICAM1 оказалось в 4 раза выше у больных ЭАГ с генотипом СС. С помощью дисперсионно- го анализа Крускела–Уоллиса определено влияние на транскрипционную активность указанного гена генотипа по rs2228145 (A > C), что говорит о его роли в патогенезе эндотелиальной дисфункции и эссенциальной артериальной гипертензии.

## Introduction

Essential arterial hypertension (EAH) is characterized by constantly elevated systolic and diastolic blood pressure (above 140/90 mm Hg). It is accompanied by chronic low-grade systemic inflammation with an increase in proinflammatory proteins in blood plasma and vascular tissues (Bautista et al., 2005). Among them, IL-6 makes a significant contribution to the pathogenesis of this disease. It can stimulate the production of acute-phase proteins and enhance the adhesive potential of vascular endothelial cells and transendothelial leukocyte migration (Virdis et al., 2014). The high IL-6 level causes an increase in the production of reactive oxygen species and alters the levels of atherogenic and antiatherogenic lipid fractions and their oxidized forms in plasma, thereby contributing to atherogenesis. This cytokine, along with other proinflammatory proteins, contributes to a decrease in the endothelial nitric oxide synthase activity, which leads to a decrease in the production of nitric oxide by vascular endothelium and disturbs the vasomotor functions of the cardiovascular system (Didion, 2017).

Interleukin 6 exerts its biologic effects through interaction with either transmembrane receptor mbIL-6R or soluble receptor sIL-6R, which are associated with another component of the receptor complex, glycoprotein 130 (GP130) (Wolf et al., 2014). The pathway for the signal from the ligand to cell via interaction with mbIL-6R is considered classical, and it is present only in a limited number of cell types, e. g. in hepatocytes, macrophages, neutrophils, and T lymphocytes. The signal transduction via sIL-6R is implemented in all types of cells and is called the trans-signaling pathway. The concentration of sIL-6R in plasma and tissues undergoing inflammation increases due to enhanced proteolytic cleavage of the membrane-bound form of the receptor by ADAM metalloproteases, while the ratio between mbIL-6R and sIL-6R changes (Wolf et al., 2014).

Some mutations in the IL6R gene also affect the balance of membrane-bound and soluble forms of the IL-6 receptor (Rafiq et al., 2007; Ferreira et al., 2013). In particular, a single nucleotide substitution (A > C) in exon 9 of the IL6R gene (rs2228145) leads to the replacement of alanine for asparagine at position 358 of the amino acid sequence of the protein and affects the ectodomain shedding process by modifying the site of polypeptide chain cleavage by ADAM10 and ADAM17 proteases and by the formation of different mRNA splice forms of this gene (Galicia et al., 2004; Rafiq et al., 2007; Ferreira et al., 2013). In C allele carriers, sIL-6R concentration is higher than in A carriers (Galicia et al., 2004; Rafiq et al., 2007; Ferreira et al., 2013). It has been shown that carriers of the C allele of rs2228145 are at lower risk of coronary heart disease development (The Interleukin 6 receptor…, 2012). However, no information on the relationship between the carrier status of allelic variations in the indicated polymorphic variant and the development of arterial hypertension has been reported hitherto.

IL-6 contributes to stable hypertension by affecting vascular remodeling and endothelial function (Didion, 2017). The IL-6/sIL-6R complex is involved in controlling vascular permeability. By acting on fibroblasts and inducing the production of vascular endothelial growth factor (VEGF), IL-6 can activate endothelial cells (Nakahara et al., 2003). These cells, activated by intercellular adhesion molecules, bind leukocytes from the bloodstream, and their transendothelial migration increases (Cook-Mills et al., 2011). Moreover, infiltration of monocytes with proinflammatory properties (Wenzel et al., 2011) is essential for the development of arterial hypertension and impaired vascular function. Once in the vascular intima, monocytes differentiate into macrophages with the proinflammatory M1 or antiinflammatory M2 phenotypes, depending on the microenvironment (i. e. concentrations of chemokines and cytokines). Polarization of macrophages to the M1 phenotype promotes atherosclerotic processes in the walls of blood vessels, which, in turn, plays an important role in establishing high blood pressure (Moss, Ramji, 2016). These processes are associated with endothelial dysfunction development, and higher levels of intercellular adhesion molecules on the surface of endothelial cells and in plasma are a marker of this process (Sprague, Khalil, 2009).

Plasma IL-6 levels are shown to positively correlate with E-selectin, vascular cell adhesion molecule-1 (VCAM-1), and intercellular adhesion-1 molecule (ICAM-1) on the surface of endothelial cells (Weiss et al., 2013). Under normal physiological conditions, vascular endothelium does not interfere with immune cell circulation in the bloodstream. This process is impaired by inflammation. Leukocytes begin to interact with endothelial cells, and this interaction ultimately leads to their tight adhesion to the endothelium surface, infiltration, and accumulation in vascular intima. The step of leukocyte attachment to the endothelium surface is characterized by interaction of integrins with ICAM-1 and VCAM-1, which are members of the immunoglobulin superfamily (Weiss et al., 2013). This process is activated when plasma levels of cytokines, reactive oxygen species, and oxidized low-density lipoproteins increase or when blood flow grows (Weiss et al., 2013).

As already noted, rs2228145 affects the level and biological activity of interleukin 6 (Rafiq et al., 2007) which acts as one of the factors enhancing transcription of ICAM1 and VCAM1 intercellular adhesion genes (Cook-Mills et al., 2011). IL-6 induces the expression of genes for intercellular adhesion molecules through activation of the IL-6/STAT3 (Signal Transducers and Activators of Transcription3) signaling pathway (Wei et al., 2018). In addition, the IL-6/sIL-6 complex triggers a signal for degradation of the nuclear factor kappa B inhibitor/ nuclear factor kappa B (IκBα/NF-κB) complex through JAK3 kinase, upregulating genes for proinflammatory cytokines and intercellular adhesion molecules. Thus, modulation of the activity of IL-6 by increasing the level of soluble receptors can affect significantly the expression of intercellular adhesion molecules and the strength of the immune response to inflammatory stimuli. In this regard, the aim of our work was to study the relationship of the carriership of allelic variations in rs2228145 with the development of essential arterial hypertension (types I and II) and the transcript levels of the VCAM1 and ICAM1 genes.

## Materials and methods

We genotyped 152 samples of DNA isolated from venous blood of patients with EAH stages I or II (80 men and 72 women) and 148 samples of DNA isolated from blood of healthy donors (65 men and 83 women). The material for the study was obtained with the assistance of the Department of Theoretical Therapy, Phthisiology, Infectious Diseases, and Epidemiology of the Medical Institute of the Petrozavodsk State University and the Clinical Diagnostic Laboratory of the Emergency Hospital, Petrozavodsk. The diagnosis of EAH was made based on the clinical recommendations of the Russian Society of Cardiology (Diagnosis and treatment…, 2010). The average age of donors from the control group was 42.5 ± 10.6 years; patients with EAH, 45.7 ± 13.1 years. The entry criteria for donors of the studied groups were as follows: the presence of informed consent, the residence in the Republic of Karelia. The general criteria for withdrawal of donors of the studied groups: the cases of infectious and inflammatory diseases in the previous month, pregnancy and lactation, smoking, diabetes, and body mass index ≥30 kg/m^2^.

DNA was isolated from peripheral blood on microcolumns using the K-Sorb kit (Synthol, Russia). The quality and quantity of DNA were assessed using a SmartSpec spectrophotometer (Bio-Rad, USA). Genotyping was performed by PCR-RFLP. The conditions for PCR-RFLP analysis are described in Table 1. The primers were synthesized at Syntol, Russia. The primers were designed with the use of Beacon Designer 5.0 software. After digestion (Hinf I (1 u), 37 °C, 3 hours), DNA fragments were separated in a 1.5 % agarose gel using TAE buffer. In some donors selected at random, the levels of ICAM1 and VCAM1 gene transcripts in peripheral blood leukocytes (PBL) were determined. To this end, venous blood samples were collected prior to prescription of antihypertensive and anti-inflammatory drugs to patients.

Levels of ICAM1 and VCAM1 gene transcripts were measured in 36 blood samples of EAH patients (stages I– II) aged 42.42 ± 2.3 years and 40 blood samples of control group donors aged 39.82 ± 3.9 years. Total RNA was isolated from PBL using the Extract RNA kit (Evrogen, Russia). The amount of total RNA was determined by use of SmartSpecPlus spectrophotometer (Bio-Rad, USA). Total RNA was treated with DNase (1 u) (SibEnzyme, Russia). The MMLV RT kit (Evrogen, Russia) was used for the first DNA chain synthesis. The quantity and quality of the isolated cDNA were analyzed spectrophotometrically using SmartSpecPlus (Bio-Rad, USA). The levels of ICAM1 and VCAM1 gene expression were evaluated by real-time PCR performed in an iCycler iQ5 (Bio-Rad, USA) using a Screen-Mix SYBRGreen kit (Evrogen, Russia). The GAPDH and 18S rRNA genes were used as reference ones. The primer sequences are shown in Table 1. The specificity of amplification products was checked by melting PCR fragments. PCR efficiency (98 %) was evaluated with a standard curve. The levels of gene transcripts were calculated by ΔΔСt (Livak, Schmittgen, 2001). Each PCR experiment was performed in at least three replications.

**Table 1.**
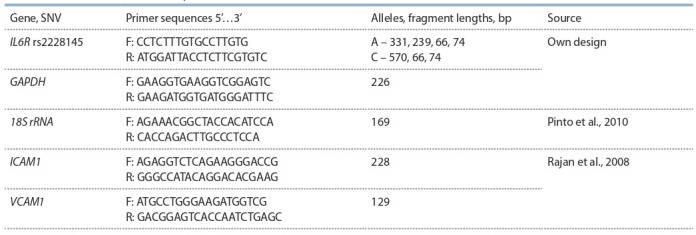
Primers for PCR-RFLP analysis and real-time PCR

The study was approved by the Medical Ethics Committee of the Ministry of Health and Social Development of the Republic of Karelia and the Petrozavodsk State University. Statistical evaluation of the data was carried out with Statgraphics 2.1 software. The significance of differences in allele and genotype frequencies between the groups was evaluated by the χ2 test. Deviations of biochemical parameters from the normal distribution were found (the Kolmogorov–Smirnov test was used, p < 0.05); therefore, the nonparametric Mann– Whitney U-test was applied to analyze the significance of differences in these indicators between the groups. The effect of genotypes on the level of transcripts was assessed with the Kruskal–Wallis test. To assess the risk of EAH development, the odds ratio (OR) was calculated with 95 % confidence interval (95 % CI) (Fletcher et al., 1998). Transcript levels are presented as mean values ± SEM. Differences were considered significant at p < 0.05.

## Results

**Fig. 1.**
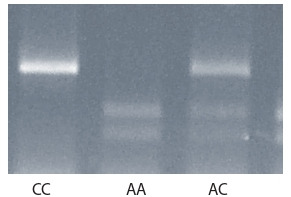
Electrophoretic image of PCR fragments of the IL6R gene in 1.5 % agarose gel after treatment with Hinf I restriction endonuclease. Allele A corresponds to DNA fragments of 331 and 239 bp; allele C, 570 bp.

We identified the following genotypes of the IL6R gene (rs2228145) in the groups: AA, AC, and CC (Fig. 1).

The genotypic data were tested for correspondence to the Hardy–Weinberg equilibrium. There was no deviation of genotype frequencies from the Hardy–Weinberg equilibrium in the healthy people group (χ2 = 1.96, df = 2, p = 0.376). In the EAH group, genotype frequencies deviated from the Hardy–Weinberg equilibrium (χ2 = 7.02, df = 2, p = 0.093).

The distributions of rs2228145 (A > C) alleles in patients with hypertension and in the control group did not differ (Table 2). The frequencies of genotypes in the studied groups were different. The frequency of the CC genotype for the rs2228145 marker of the IL6R gene in patients with EAH was significantly higher than in the control group.

**Table 2.**
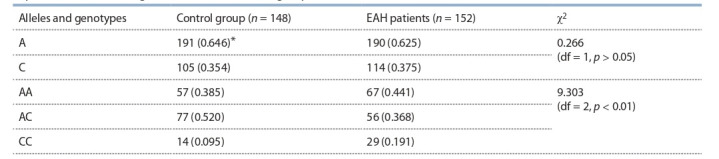
Alleles and genotypes distribution for A > C rs2228145 polymorphic marker of IL6R gene in patients with EAH (stages I–II) and in the control group * The frequencies are indicated in parentheses.

According to the odds ratio, individuals with the CC genotype for rs2228145 have a 2.2-fold higher risk of EAH development than the carriers of other genotypes (Table 3).

**Table 3.**
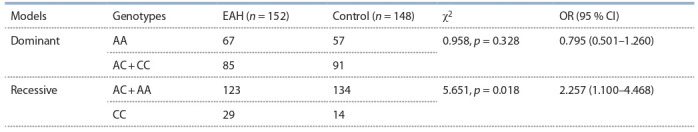
Dominant and recessive models of rs2228145 (A > C) genotype distributions of the IL6R gene

It was found that patients with EAH had higher levels of gene expression of adhesion molecules VCAM1 and ICAM1 in PBL ( p = 0.005 and p = 0.0006, respectively) compared to donors from the control group.

**Fig. 2.**
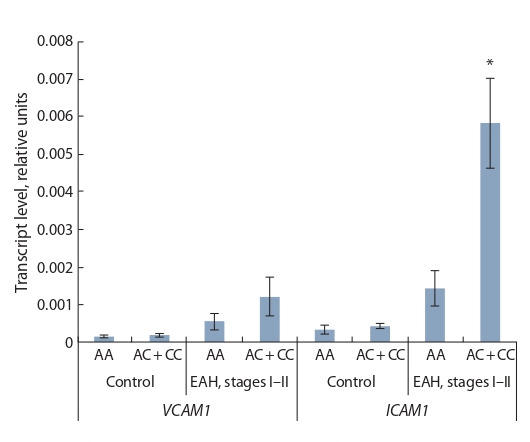
The levels of VCAM1 and ICAM1 gene transcripts in PBL of the healthy people and the patients with EAH depending on rs2228145 allelic variations of the IL6R gene. * The differences are significant when compared with carriers of genotype АА ( р = 0.021).

The VCAM1 mRNA levels in PBL of individuals with the rs2228145 genotypes AA and AC + CC of the IL6R gene differed insignificantly in either controls or the group of patients with EAH ( p = 0.292 and p = 0.0710, respectively) (Fig. 2). We found differences in ICAM1 gene mRNA levels in patients with EAH with different rs2228145 allelic variations of the IL6R gene (see Fig. 2). In individuals with C allele, the level of ICAM1 gene transcripts was four times higher than in individuals with alternative variants for the polymorphic marker in question. An effect of rs2228145 genotype on the ICAM1 gene mRNA levels was revealed (H = 4.74, p = 0.029, respectively).

## Discussion

We have found an association between rs2228145 allelic variations of the IL6R gene and EAH development in humans. Carriers of the CC genotype for this marker are characterized by 2.3-fold higher risk of EAH. Interestingly, it has been reported that the C allele is protective against the development of coronary heart disease and rheumatoid arthritis (Jiang et al., 2010; Sarwar et al., 2012; Ferreira et al., 2013). This fact can be explained by a decrease in mbIL-6R expression on the surface of T lymphocytes and monocytes, leading to a weakening of the classical IL-6 signaling pathway, as apparent from the depletion of phosphorylated forms of transcription factors STAT1 (Signal Transducers and Activators of Transcription1) and STAT3 in these cells (Ferreira et al., 2013). The pathogenetic effect of the C allele for the discussed polymorphic marker on EAH progress may be due to the fact that its presence results in a higher level of soluble IL-6 receptors in plasma (Galicia et al., 2004; Rafiq et al., 2007; Ferreira et al., 2013) and secures more stable IL-6 signaling. Approximately 70 % of secreted IL-6 is known to bind to sIL-6R in blood (Gaillard et al., 1999). Thus, sIL-6R acts as a carrier molecule for IL-6. In vascular endothelial cells this cytokine exerts its effects through the IL-6/sIL-6R signaling pathway. The level of sIL-6R in plasma increases in patients with cardiovascular diseases (Sarwar et al., 2012). In turn, this can lead to additional activation of signaling pathways transducing the inflammatory signal in endothelial cells in individuals with the AC and CC genotypes for rs2228145. It is known that the IL-6/sILR-6 complex activates p65 NF-κB and STAT3 transcription factors and contributes to the increase in mRNA levels of the IL6, GP130, and STAT3 genes (Kim et al., 2011), thereby enhancing local and systemic inflammatory responses.

Proinflammatory cytokines, such as TNFα and IL-6, enhance the transcriptional activity of adhesion molecule genes (VCAM1 and ICAM1) (Sprague, Khalil, 2009). The transmission of the TNFα and IL-6 signals to the nuclear apparatus of cells is initiated through the regulation of NADPH oxidase 2 activity and the production of reactive oxygen species (Cook- Mills et al., 2011; Wang et al., 2016). An increase in the content of ICAM-1, VCAM-1, and E-selectin on the surface of endothelial cells and in plasma is associated with the risk of coronary heart disease (Belokopytova et al., 2013), atherosclerosis (Galkina, Ley, 2007), and pulmonary hypertension (Kato et al., 2005). Therefore, the modulation of interleukin 6 activity by changing the ratio of membrane-bound and soluble forms of its receptors, determined by mutations that affect ectodomain shedding, may affect the expression level of intercellular adhesion molecules significantly. Indeed, we found that the rs2228145 genotype affects the transcriptional activity of the ICAM1 gene in peripheral blood leukocytes. The data obtained may indirectly point to alteration of ICAM1 transcriptional activity in endothelial cells and, highly likely, to the development of endothelial dysfunction. Thus, the presence of the C allele for the rs2228145 marker of the IL6R gene may be responsible for the high level of ICAM1 mRNA in EAH patients, which is likely to contribute to the pathogenesis of endothelial dysfunction and EAH development.

## Conflict of interest

The authors declare no conflict of interest.
